# Effect of Chronic Apical Periodontitis in Primary Molars on Succedaneous Permanent Teeth: An Observational Retrospective Study

**DOI:** 10.7759/cureus.45275

**Published:** 2023-09-14

**Authors:** Nileshrao Patil, Aruna P Vishwakarma, Romalpreet Singh, Monika Aggarwal, Mohd Osman Ali, Abdul Rahim Khan

**Affiliations:** 1 Department of Conservative Dentistry and Endodontics, Jawahar Medical Foundation's (JMF) Annasaheb Chudaman Patil Memorial (ACPM) Dental College, Dhule, IND; 2 Department of Pediatric Dentistry, Jawahar Medical Foundation's (JMF) Annasaheb Chudaman Patil Memorial (ACPM) Dental College, Dhule, IND; 3 Department of Conservative Dentistry and Endodontics, Desh Bhagat Dental College, Malout, IND; 4 Department of Pediatric Dentistry, Sudha Rustagi College of Dental Sciences and Research, Faridabad, IND; 5 Department of Orthodontics, Sharavathi Dental College and Hospital, Shivamogga, IND; 6 Department of Orthodontics, Mithila Minority Dental College and Hospital, Darbhanga, IND

**Keywords:** malformation, permanent successor, apical peridontitis, primary teeth, maturation

## Abstract

Introduction: Periapical infection of primary molars affects the development of permanent teeth (premolars). Therefore, the present study was conducted to test the null hypothesis in children aged 4-10 years with chronic apical periodontitis (CAP) of the primary molars.

Materials and methods: A retrospective, cross-sectional study was conducted on 185 panoramic radiographs of healthy children aged 4-10 years with CAP in the primary molars. A total of 256 infected primary molars (144 teeth in females, 112 teeth in males) were analyzed, radiographically, and compared with 245 healthy primary molars on the contralateral side. Permanent successors were evaluated for follicular damage, maturation, morphology, and deviation in the eruption path. Primary molars were evaluated for root resorption. Sixteen permanent teeth on the affected side and five teeth on the control side were excluded due to abnormal development. Student’s t-test and the chi-square test were used to analyze the data.

Results: The null hypothesis is rejected. There were significant differences in the developmental status of permanent successors on the affected side, compared to the normal side at four to seven years (p<0.05). There were no significant sex differences in the abnormalities of permanent successors on the affected side (p>0.05). As the root resorption of the primary molars increased, the follicular damage observed in the permanent successors also increased (p<0.05), which suggests that, as the infection of primary molars increases, more damage is caused to underlying permanent successors (premolars).

Conclusion: Apical periodontitis of the primary molars retards the development of permanent successors (premolars), affects their shape, causes follicular damage, and alters the eruption path.

## Introduction

In recent years, there has been an increasing demand for oral care in the pediatric population, and treatment is usually required for pain management due to caries, apical periodontitis, or traumatic injuries. Chronic apical periodontitis (CAP), which is the most common cause of a patient’s visit to a pedodontist, is characterized by pathogenic resorption of bone around the root apex of the teeth, resulting in primary tooth loss [1]. The impact of the condition is not limited to the primary dentition as it has the potential to result in pronounced hypoplasia, delayed or altered development, modified eruption patterns, and, in extreme cases, the development of odontogenic cysts that can lead to necrosis of the tooth germs of permanent successors [2,3].

Lo et al. conducted a study confirming that the degree of caries lesion present on a primary molar can significantly impact the likelihood of occurrence of enamel defects on the permanent successors. As the size and depth of the carious lesion in a primary tooth increases with periapical infection, the chances of enamel defect onset in the permanent successor tooth increase [4]. The potential explanation for this phenomenon could be attributed to the proximity of the permanent tooth germ to the bifurcation and apices of the primary teeth. This closeness, coupled with the existence of numerous root and accessory canals, increases the likelihood of primary tooth infection infiltrating the apical and furcation regions, thereby impinging upon the successive permanent tooth follicle [5].

According to some authors, degradation of the overlying osseous tissue due to periapical infection of the deciduous dentition expedites the emergence of succeeding permanent teeth [6]; however, other studies posit that it retards the eruption of permanent teeth [7]. The relationship between enamel hypoplasia of permanent successors and periapical infection of primary teeth is a subject of various controversies. The multifactorial etiology of enamel defects has led to several debates. While isolated enamel defects may be attributed to CAP in primary teeth, diffuse defects affecting multiple teeth may be linked to systemic causes [8].

Therefore, it is of utmost importance to address pulpal infection in primary teeth during its early stages to prevent advancement into CAP, which may harm permanent successors. The present study aimed to evaluate the effects of CAP in the primary molars on successive permanent teeth (premolars). The null hypothesis of this study proposed that CAP in primary teeth has no significant influence on the development of erupting permanent successors (premolars).

## Materials and methods

This retrospective, observational, cross-sectional study was conducted in the Department of Pedodontics, Jawaharlal Medical Foundation’s Annasaheb Chudaman Patil Dental College, from January 2021 to December 2022. Approval was obtained from the institutional ethics committee (EC/NEW/INST/2022/2959/098). Informed consent was obtained from parents or legal guardians to use their records for study purposes.

Sample size calculation

The sample size was calculated using the GPOWER statistical software (Ver. 3.1; Franz Faul, Universität Kiel, Kiel, Germany), with a type I error of 0.05 and a beta error of 0.2. The sample size was calculated as 130, with 80% power [7]. The present study was conducted on 185 panoramic radiographs, obtained from healthy children. This provided a 90% power for this study.

A total of 500 panoramic radiographs of healthy children within the age range of 4-10 years were collected from departmental records, of which 185 panoramic radiographs (90 males, 95 females) were selected based on the inclusion and exclusion criteria of the study. Of the 185 radiographs, 170 (80 males, 90 females) also served as controls, and the contralateral unaffected primary molars and underlying permanent teeth were assessed.

Inclusion and exclusion criteria

The presence of good quality radiographs, CAP of at least one primary molar involving mesial or distal or both roots, and healthy children in early mixed dentition period of age 4-10 years, with normal development, were selected for the study, irrespective of sex and socioeconomic status. Children with abnormal growth, cleft lip and palate, disorders affecting jaw growth, genetic abnormalities, systemic disorders, or multiple extracted teeth were excluded from the study.

Method of evaluation of the primary molars

Root resorption (mesial or distal or both) was evaluated in the primary molars. One point was assigned in cases where there was an absence of root resorption or when the extent of root resorption was less than one-third of the root length. Two points were allotted in cases where root resorption exceeded one-third of the root length, but the pulp floor was still present. Three points were designated for residual root, whereas early tooth loss was assigned to four points.

Method of evaluation of the permanent successors (premolars)

Maturation: The maturation status of successive permanent teeth was assessed according to Nolla’s stages of tooth calcification [9].

Follicular damage (FD): One point was assigned in cases with no follicular damage; two points were assigned in cases where damage was less than one-third of the follicle; if damage was more than one-third but less than two-third of the follicle, three points were assigned; and four points were assigned in cases where damage exceeded two-third of the follicle.

Deviation: The evaluation of any deviation in the eruption path of the permanent successor was conducted in the mesiodistal orientation. A score of 0 was assigned for the absence of any deviation, whereas a score of 1 was assigned for instances of a noticeable deviation.

**Figure 1:**
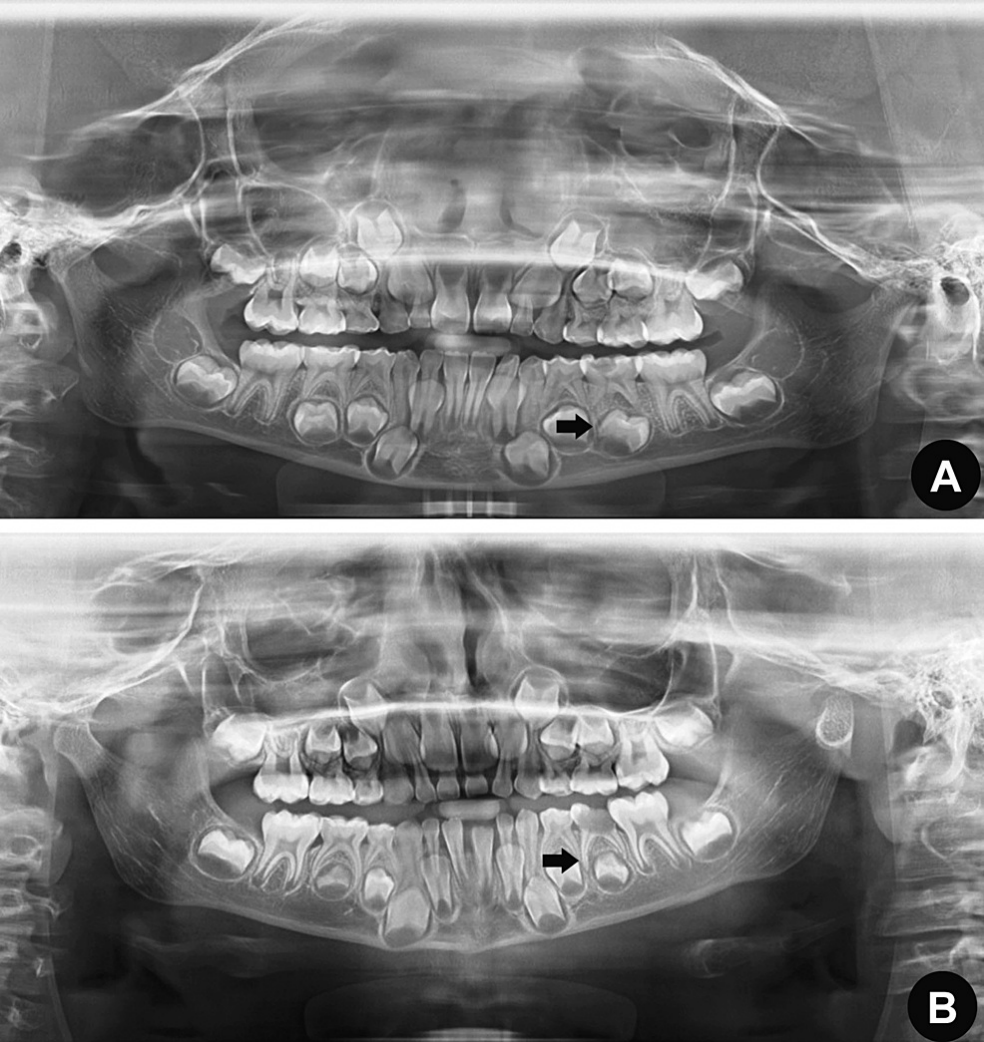
Panoramic radiograph for the evaluation of permanent successors and primary molars (A) deviation and (B) follicle damage

Malformation: If the assessment of morphology is normal, it results in zero points. Conversely, if the morphology is abnormal, this results in a score of one point (in the case of abnormal morphology of permanent teeth, its maturation status was not evaluated) (Figure 1).

Test of reliability

Two experienced dentists (AP and PG), who had more than five years of clinical experience, evaluated the radiographs. After two weeks, 30 radiographs were evaluated for intra- and inter-observer reliability using Cohen’s kappa test. High intra-observer reliability ranging from 0.92 to 0.97 and inter-observer reliability ranging from 0.88-0.94 were obtained.

Statistical analysis

The data were arranged methodically using Microsoft Excel. The collected data, as a whole, were statistically analyzed using Statistical Product and Service Solutions (SPSS) software( version 22; for Windows, Chicago, IL, USA). The Shapiro-Wilk test was used to check the normality of the data. As the data were found to be normally distributed, parametric and non-parametric tests were used for analysis. Student’s t-test was used to evaluate the differences in mean maturity scores between the control and affected teeth. A chi-square test was performed to compare the proportion of various abnormalities of the affected permanent successors and root resorption in primary molars between genders. The chi-square test was also used to correlate the follicular damage of the permanent successors and root resorption of the primary molars on the affected side. The significance level was set at p<0.05.

## Results

A total of 256 affected primary molars were evaluated using 185 panoramic radiographs. Sixteen affected permanent teeth were excluded from the study due to abnormal development (included only in malformation estimation). Hence, 240 permanent teeth were evaluated for maturity, deviation in eruption path, and follicular damage. A total of 240 unaffected contra-lateral primary molars and successive permanent teeth were evaluated as controls, after excluding five abnormal permanent teeth (included only in malformation estimation).

The null hypothesis was rejected in our study because there were statistically significant differences between the control and the affected sides. The rate of development, as evident from Nolla’s stages of tooth calcification, revealed that tooth development of permanent successors on the affected side was delayed by four to six months compared to that on the normal side (negative sign for mean difference) for all ages. It was statistically significant for ages four to seven years (p<0.05) and non-significant for 8-10 years (p>0.05), as shown in Table 1.

**Table 1:** Maturation scores and the mean difference of permanent successors on affected and control sides for each age *p-value<0.05: Significant; SD, Standard deviation

Maturation age (years)	No. of teeth	Mean±SD (affected)	Mean±SD (control)	Mean difference	p-value
4	48	2.46±0.23	3.16±0.72	-0.70	0.001*
5	44	3.22±0.45	3.95±0.37	-0.73	0.003*
6	28	4.21±0.34	4.81±0.67	-0.61	0.001*
7	40	4.13±0.69	4.89±0.57	-0.76	0.001*
8	28	5.03±0.97	5.21±0.78	-0.18	0.138
9	24	6.83±0.67	6.96±0.34	-0.13	0.231
10	28	6.71±0.68	6.82±0.56	-0.11	0.163

This shows that periapical infection of primary molars affects the mineralization of permanent successors in the early stages of development (before Nolla stage 5). Most of the effects were seen in children in the four to five years age group (Nolla's stages 2 and 3).

There were no statistically significant differences seen between genders, in abnormalities, such as follicular damage, malformations, deviation in the path of eruption of the permanent successors, and root resorption of the primary molars on the affected side, as shown in Table 2.

**Table 2:** Proportion of different abnormalities of permanent successors and root resorption in primary molars on the affected side in both genders

Type of abnormality	Male	Female	Total	Chi-square value	p-value
Follicle damage	34 (41.7%)	48 (58.3%)	82 (34%)	0.76	0.381
Malformation	10 (62.5%)	6 (37.5%)	16 (6.6%)	1.26	0.265
Deviation	24 (38%)	38 (62%)	62 (25.8%)	0.72	0.393
Root resorption	44 (28.7%)	112 (71.7%)	156 (65%)	3.07	0.079

**Figure 2:**
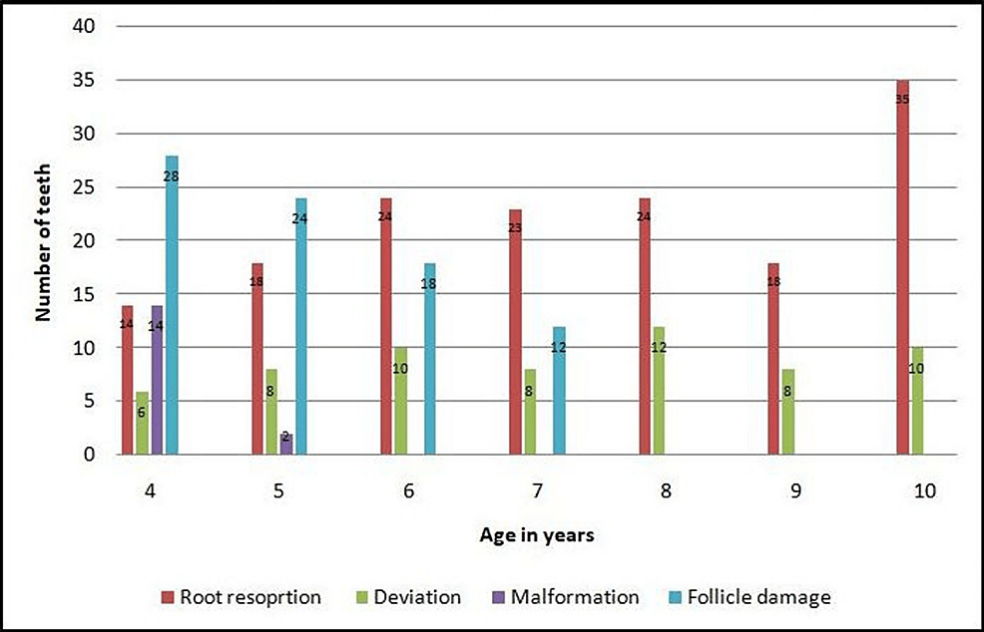
Proportion of abnormalities of permanent successors and root resorption in primary molars on the affected side in different ages

The follicular damage was mostly observed from four years until seven years of age. Malformation of the permanent successors on the affected side was mostly present at four years of age, as shown in Figure 2.

This suggests that the shape of the permanent teeth and calcification were the most affected if infection of the primary molars occurred in the initial stages of development.

**Figure 3:**
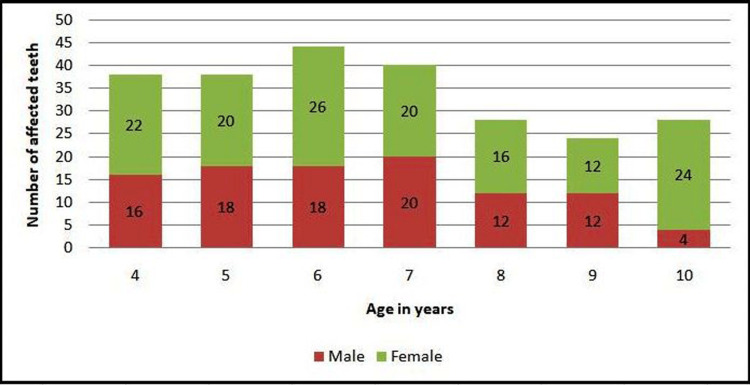
Distribution of gender for affected teeth involved in different age groups

Of the 256 affected primary molars, females had 144 infected teeth, compared to 112 infected primary molars in males. Females had a greater number of infected primary molars than males at all ages, as shown in Figure 3. This shows that apical periodontitis of the primary molars is more prevalent in females than in males.

Most primary molars with apical periodontitis had less than one-third root resorption (86 teeth had no resorption, 106 teeth had resorption of less than one-third, and 48 teeth had root resorption exceeding one-third). There was a significant difference in follicular damage with root resorption in primary molars with apical periodontitis (p<0.05). In cases with no resorption of primary molars (score 1), follicular damage was not observed in 73 out of 86 teeth (score 1), whereas as the root resorption increased to one-third (score 2), follicular damage was evident (score 2), which did not exceed one-third (58 teeth out of 106 teeth). As root resorption further increased to more than one-third (score 3), a greater number of premolars showed follicular damage, exceeding one-third (score 3) (Table 3). Cases with root resorption exceeding two-third of the root (score 4) were not present in our study.

**Table 3:** Comparison of the follicular damage (FD) in successive teeth with resorption of root in primary teeth having apical periodontitis *p-value<0.05: Significant

Root resorption	FD score 1	FD score 2	FD score 3	Chi-square value	p-value
No resorption (score 1)	73	12	1	58.95	0.0001*
1/3rd resorption (score 2)	36	58	12		
2/3rd resorption (score 3)	4	16	28		

## Discussion

The identification and diagnosis of dental abnormalities at an early stage are crucial for the assessment of pediatric patients and formulation of treatment strategies. Chronic periapical periodontitis of deciduous teeth rapidly affects the surrounding tissues. This process occurs simultaneously with proliferative and productive inflammation, and diffuses extensively and rapidly into adjacent regions as well as along the vessels into faraway areas around the permanent teeth buds. This dissemination may be attributed to the lack of resistance of the tissue surrounding the deciduous teeth and is also facilitated by the pressure exerted by the emerging bud of their successors. Pressure on the bone and soft tissue facilitates and amplifies the extension of inflammation into the sac of the buds [10].

This study was conducted in a cohort of pediatric patients aged 4-10 years. This age range was chosen because of the high probability of the predecessor teeth being present in the oral cavity. Significant differences were observed in the formation of permanent teeth (premolar) in individuals with an uninfected primary predecessor, compared to individuals with infected primary molars.

Assessment of the affected primary teeth (molars)

Root resorption was evaluated in the primary teeth, and if the resorption exceeded one-third of the root length (two points), the case was not indicated for root canal treatment [11]. In our study, most affected primary teeth scored less than two points and were, therefore, indicated for root canal treatment. An increase in the severity of infection in the primary dentition led to a corresponding increase in follicular damage sustained by the succeeding permanent teeth. This was in accordance with a previous study [12]. This might be due to the presence of inflammatory mediators, such as interleukin 1 alpha and beta (IL-1α and IL-1β, respectively), in periapical infections of the primary teeth, which are related to osteoclastic resorption, stimulating lymphocytes, and neutrophils [13]. This study also supports the notion that loss of the essential pulp of a primary dentition, irrespective of the duration of the ailment, could potentially exert an irrevocable influence on permanent dentition.

Assessment of permanent teeth (premolars)

Nolla’s stages of calcification were used to evaluate the development of succedaneous permanent teeth using panoramic radiographs, which are extensively employed worldwide and are deemed trustworthy and precise. However, this is not without its constraints [14]. With regard to determining the placement of permanent successors, the two-dimensional nature of the panoramic film precludes accurate determination of buccal-lingual displacement.

In the present study, 240 premolars on the affected side were compared with their counterparts on the unaffected side. The observed phenomenon revealed that the process of calcification in the affected teeth was delayed compared with that in the unaffected permanent teeth. These findings indicate that CAP in the primary teeth can potentially impede the growth and maturation of permanent successors. Our findings are in agreement with prior studies that indicated retardation in the maturation of permanent successors due to periapical infection of the primary teeth overlying them [3,7,13]. This difference was most evident in children of four to six years, in both males and females. The probable reason for this finding might be due to the fact that developmental disturbances in calcification are most evident at the initial stages of crown mineralization (Nolla’s stages 2 and 3).

Our study also revealed a deviated path of eruption in the affected permanent teeth compared to that in the unaffected teeth. The present findings suggest that CAP of the primary dentition exerts a significant impact on the orientation of the emergence of permanent successors, which is consistent with prior investigations [2,7]. The potential explanation for this observation could be attributed to the obliteration of the gubernacular canal caused by the progression of the infection into the periradicular bone [3]. The findings of our study indicate that the highest occurrence of anomalous positioning and morphological abnormalities was observed in patients aged less than eight years [7]. This suggests that, once the teeth have undergone crown formation and calcification, as evidenced by the Nolla stage 6, the impact of CAP on permanent successors is significantly diminished. Furthermore, the impact of this condition is closely linked to the developmental stage of the permanent successors. Several experts have highlighted that permanent successors with fully formed crowns, as indicated by Nolla's stage 6, remain unaffected by inflammation arising from abscesses or cysts in the primary molars or their corresponding extractions [15]. The results of this study support this view.

Abnormalities in the shape of the permanent teeth were noticed on the affected side compared to the normal side, and these abnormalities were noticed mostly in four-year-old children, who had a history of infected, unrestored primary molars at the age of 2.5 to three years. This may be related to the effect of periapical infection of the primary molar on the follicle of the succedaneous permanent tooth. This effect is more pronounced when the underlying permanent tooth is at the formation stage (cap, bell, or secretory stage of the hard-tissue matrix). Therefore, infection of primary molars after four to five years of age does not affect the morphology of the underlying premolars, as they have already begun their crown mineralization process [3].

The potential explanation for the disparate outcomes documented in the existing literature may stem from the failure to account for confounding variables such as sex and matched control group, as well as an inadequate sample size for an observational study. This study was conducted using 185 radiographs of 256 affected teeth, which provided a power of 90%. The control group included unaffected contralateral teeth of the same patient, eliminating bias due to individual variations. The study was conducted with nearly equivalent numbers of male and female participants.

Clinical implications of the study

As chronic periapical periodontitis of the primary teeth alters the path of eruption, causes follicular damage, and affects calcification of the underlying permanent teeth, dental practitioners should exhibit a heightened level of awareness regarding the influence of periapical disease that afflicts primary teeth and its concomitant effects on the subsequent permanent dentition. It is incumbent upon healthcare providers to implement a more robust and comprehensive monitoring regimen for patients afflicted with this condition, with particular emphasis on closely scrutinizing the development and emergence of permanent successors and promptly modifying treatment plans and interventions, as necessary. In cases where there is definitive evidence of a substantial deleterious impact on the permanent teeth, it is vital to expeditiously extract these compromised teeth rather than retain them in a carious state.

Limitations of the study

It is imperative to recognize that the study's retrospective design imposed limitations on our ability to manipulate variables within the dataset, resulting in the need to address the heterogeneity among the groups. Panoramic radiographs, which are two-dimensional in nature, were used in our study; hence, more advanced diagnostic three-dimensional aids, such as cone beam computed tomography (CBCT), should be used. Future prospective, multicentre, longitudinal studies should be conducted using CBCT.

## Conclusions

CAP of the primary dentition significantly influences the eruption path of successive permanent teeth. As the infection of the primary dentition intensified, the underlying permanent dentition also experienced increased follicular damage. Furthermore, in contrast to the unaffected dentition, maturation of the permanent dentition on affected side was significantly retarded. Hence, it is of utmost importance to perform conservative therapy on the primary teeth prior to the onset of irreversible pulp inflammation and subsequent periapical infection.
